# A topical rectal douche product containing Q-Griffithsin does not disrupt the epithelial border or alter CD4^+^ cell distribution in the human rectal mucosa

**DOI:** 10.1038/s41598-023-34107-w

**Published:** 2023-05-09

**Authors:** Mathias Franzén Boger, Nora Benhach, Tyra Hasselrot, Rhonda M. Brand, Lisa C. Rohan, Lin Wang, Ian McGowan, Stacey Edick, Ken Ho, Leslie Meyn, Nobuyuki Matoba, Kenneth E. Palmer, Kristina Broliden, Annelie Tjernlund

**Affiliations:** 1 Division of Infectious Diseases, Department of Medicine Solna, Center for Molecular Medicine, Karolinska University Hospital, Karolinska Institutet, Bioclinicum J7:20, 171 64 Solna, Sweden; 2 Magee Womens Research Institute, Pittsburgh, PA USA; 3 Department of Medicine, University of Pittsburgh School of Medicine, Pittsburgh, PA USA; 4 Department of Pharmaceutical Sciences, University of Pittsburgh School of Pharmacy, Pittsburgh, PA USA; 5 Orion Biotechnology, Ottawa, Canada; 6 Center for Predictive Medicine for Biodefense and Emerging Infectious Diseases, University of Louisville, Louisville, KY USA; 7 UofL Health-Brown Cancer Center, University of Louisville, Louisville, KY USA; 8 Department of Pharmacology and Toxicology, School of Medicine, University of Louisville, Louisville, KY USA

**Keywords:** HIV infections, Imaging the immune system, Mucosal immunology, Drug discovery

## Abstract

To reduce HIV transmission, locally applied pre-exposure prophylaxis (PrEP) products for anorectal use will be important complements to oral and injectable PrEP products already available. It is critical to preserve an intact rectal epithelium and avoid an influx of mucosal HIV target cells with such product use. In this phase 1 clinical trial, we evaluated application of a topical rectal douche product containing Q-Griffithsin (Q-GRFT). Colorectal tissue samples were obtained via sigmoidoscopy at baseline, 1 and 24 h after single-dose exposure in 15 healthy volunteers. In situ staining for epithelial junction markers and CD4^+^ cells were assessed as an exploratory endpoint. A high-throughput, digitalized in situ imaging analysis workflow was developed to visualize and quantify these HIV susceptibility markers. We observed no significant differences in epithelial distribution of E-cadherin, desmocollin-2, occludin, claudin-1, or zonula occludens-1 when comparing the three timepoints or Q-GRFT versus placebo. There were also no differences in %CD4^+^ cells within the epithelium or lamina propria in any of these comparisons. In conclusion, the rectal epithelium and CD4^+^ cell distribution remained unchanged following topical application of Q-GRFT. In situ visualization of HIV susceptibility markers at mucosal sites could be useful to complement standard product safety assessments.

## Introduction

Approximately 84 million people have been infected with HIV since the beginning of the HIV epidemic, and approximately 38.4 million were living with HIV at the end of 2021^1^ Implementation of medication regimens, such as pre-exposure prophylaxis (PrEP) against HIV infection, has been a successful preventive strategy for some risk groups. However, implementing optimal and timely treatments that prevent mucosal viral entry and replication at the time of sexual intercourse are crucial.

Receptive anal intercourse (RAI) is associated with the highest risk for acquiring HIV^2^. This may be at least partly attributed to the fragility of the rectal epithelium, which consists of a single layer of cells connected by epithelial junction proteins (EJPs) that under healthy conditions form a protective barrier against HIV and other pathogens^3^. Consequently, the risk of tears in the rectum is high, and these tears will facilitate entry of pathogens including HIV. Mucosal trauma, a non-optimal microbiome, preexisting inflammation, and the presence of other sexually transmitted infections (STIs) can also contribute to an increased risk of acquiring HIV^4,5^.

Novel strategies that improve prevention against HIV infection are necessary to counter the HIV epidemic. Preferences for HIV prevention products vary across groups and geographic locations. While HIV PrEP clearly reduces transmission among at-risk individuals, uptake and efficacy can be variable. Oral emtricitabine/tenofovir disoproxil fumarate (Truvada) has been approved for use prior to RAI. Antiviral products for rectal use (microbicides) in the form of gels, suppositories, enemas or douches are other options that have been tested in clinical trials^6^. The development of a 1% formulation of tenofovir gel is promising and appears to be both safe in men and women following daily rectal application, or application prior to and after RAI^7–10^.

Griffithsin (GRFT) is a naturally occurring lectin protein originally isolated from the red alga Griffithsia^11,12^. To produce a more stable compound that is less prone to oxidation, GFRT has been genetically modified with one amino acid replacement from methionine to glutamine (Q) to create Q-GRFT^13^ Both GRFT and Q-GRFT are some of the most potent broad-spectrum antivirals ever tested, and its activity is under study for potential therapeutic applications against several sexually transmitted viral pathogens, including HIV, herpes simplex virus type-2 (HSV-2), human papillomavirus and hepatitis C virus (HCV)^14^. GRFT has also proven effective against Candida infections^15,16^ and a Q-GRFT nasal spray has recently been tested in a first-in-human clinical study as a prophylaxis modality against SARS-CoV-2^17,18^. Being a macromolecule of non-human origin GRFT is not indicated for use as an oral antiretroviral therapy and thus differs from other PrEP strategies, which can select for viral resistance and therefore compromise ART^19^. GRFT can be formulated into a number of delivery vehicles for topical administration, including gels, films, suppositories, enemas or douches, and rings^11^. Both GRFT and Q-GRFT are now investigational drug candidates developed as vaginal and rectal antiviral agents, respectively, against HIV infection^20–22^.

In this study, we analyzed rectal biopsies from the PREVENT study (NCT04032717), a phase 1 clinical trial evaluating the safety and acceptability of the rectal application of a new anti-HIV rectal douche product containing Q-GRFT^23^. Our exploratory aim was to investigate the effects of Q-GRFT on the human rectal mucosa in vivo by assessing a panel of epithelial junction proteins, in addition to CD4^+^ cell distribution. Stable expression of these biomarkers indicate maintained integrity of the epithelial barrier and a non-inflammatory environment. We thus assessed E-cadherin, an adherens junction protein located on the surface of adjacent cells, which acts as a marker of epithelial integrity because of its adhesive properties maintaining cell to cell contact. Additionally, we examined expression levels of desmocollin-2, a desmosomal protein that acts as an adhesion molecule similar to E-cadherin. The tight junction proteins occludin and claudin-1, as well as their anchoring protein zonula occludens-1 (ZO-1), which connects tight junction proteins to the actin cytoskeleton, were also assessed. Moreover, CD4^+^ cells were analyzed, not only as target cells for HIV but also as markers of inflammation to reflect leukocyte influx.

## Methods

### Ethics declaration

This study was performed following approval from the Institutional Review Board at the University of Pittsburgh (PRO 19030322), and the Regional Ethical Review Board of Stockholm (Dnr. 2016/2561-31/12). Written informed consent was obtained from all study participants. This study was performed in accordance with the declaration of Helsinki.

### Description of study and enrolled patients

A total of 18 individuals were enrolled in the PREVENT study, a phase 1 clinical trial performed at the University of Pittsburgh, PA, USA (ClinicalTrials.gov Identifier: NCT04032717).

Eligible participants were healthy, cisgender males or females, with a minimum age of 18 years. They had to be HIV seronegative with a reported history of RAI at least five times in their lifetime (once in the past year) and willing to use an effective method of contraception throughout the study. Females were not pregnant or breastfeeding and had a regular menstrual cycle. Individuals with abnormalities of the colorectal mucosa, significant gastrointestinal symptoms (such as a history of rectal bleeding) were excluded from the study. Moreover, individuals with evidence of anorectal *Chlamydia trachomatis* or *Neisseria gonorrhoeae* infection, syphilis, active HSV lesions, chancroid, genital sores or ulcers, or symptomatic genital warts requiring treatment, chronic hepatitis B or hepatitis C antibody positivity, or a requirement to use drugs that were likely to increase the risk of bleeding following mucosal biopsy were also excluded from the study. Participants had baseline safety laboratory evaluations at study enrolment that included complete blood count, creatinine, alanine aminotransferase and aspartate aminotransferase.

**Figure 1:**
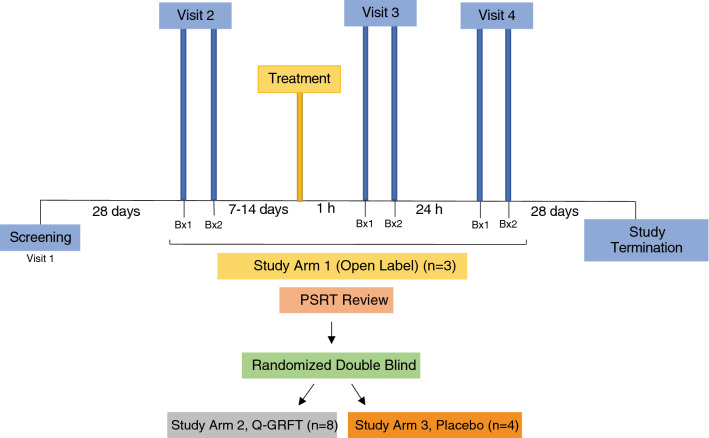
Schematic display of the clinical trial. Study arm 1 was an open-label study in which all participants were treated with Q-GRFT (n = 3). This was followed by a randomized, blinded trial, in which study arms 2 and 3 were treated with Q-GRFT (n = 8) and placebo (n = 4), respectively. Abbreviations: Bx1, biopsy 1; Bx2, biopsy 2; PSRT, Protocol Safety Review Team.

An outline of the study is presented in Fig. 1. At visit 1, participants were screened to exclude those with anorectal STIs. Up to 28 days after screening, eligible participants returned for a baseline (BL) visit (visit 2). Flexible sigmoidoscopy was performed to obtain BL colorectal biopsy samples. Participants returned 7–14 days following BL and received a clinician-administered single-dose of Q-GRFT, followed by collection of colorectal biopsies at 1 h post-application (PA) (visit 3). Participants returned to the clinic the subsequent day (visit 4) for collection of colorectal biopsies at 24 h PA. These timepoints were selected based on historical experience of our group^24^ and the desire to not perform flexible sigmoidoscopies with extensive biopsy removal more frequently than every 24 h for the comfort of the patients. This interval also permitted both early and late timepoints with respect to anticipated Q-GRFT tissue levels. A final study exit telephone call was conducted within 1 week after the last study visit to assess safety issues. Fifteen of the 18 study participants completed the full study, including undergoing all 3 sigmoidoscopies. Two participants withdrew after visit 2 and one participant after visit 3.

The first 3 study participants were assigned to study arm 1 (open-label Q-GRFT). These 3 participants received a clinician-administered single dose of the rectal douche containing 40 mg Q-GRFT. Each douche was compounded by a pharmacist prior to administration by diluting Q-GRFT drug substance (9.6 mg/mL in phosphate buffer pH7.4) with 0.9% normal saline to form 0.32 mg/mL of Q-GRFT in 125 mL. Once the participants in study arm 1 completed visit 4, the Protocol Safety Review Team (PSRT) conducted an interim review of the clinical and laboratory data. As no significant safety concerns were observed, the PSRT approved enrollment in study arms 2 and 3. Thus, the remaining 12 participants were assigned in a randomized and blinded fashion to either study arm 2 (Q-GRFT, n = 8) or study arm 3 (placebo, n = 4), representing a 2:1 Q-GRFT:placebo ratio. Patients in study arm 2 received the same Q-GRFT treatment as those in arm 1. Participants in study arm 3 received placebo rectal douche (0.9% sodium chloride solution). In total 90 rectal biopsy samples (2 samples from each participant at each collection time) were collected.

### In situ staining of rectal tissue samples

Three sets of dual immunofluorescence staining were performed on 8-µm thick sections of cryopreserved rectal biopsies to assess the expression of CD4 and the selected EJP markers. Assessment of E-cadherin and CD4 was performed on both biopsies from each participant and timepoint and an average per participant was calculated. For the remaining EJPs, only one biopsy was used per participant and timepoint. All tissue sections were air-dried for 1 h at room temperature, fixed in 100% methanol for 10 min, and then air-dried for 1 h. Phosphate-buffered saline containing 1% HEPES (HyClone, Nordic Biolabs, Täby, Sweden) and 0.1% Saponin (Sigma Aldrich, Solna, Sweden) was used as the washing buffer between each incubation step.

Samples were first incubated with Alexa Fluor 647–conjugated mouse anti–E-cadherin antibody (clone: 36, BD Biosciences, Stockholm, Sweden), followed by the addition of rabbit anti-CD4 antibody (clone: EPR6855, Abcam, Cambridge, UK) and donkey anti-rabbit Alexa Fluor 555–conjugated secondary antibody (A31572, Invitrogen, Thermo Fischer Scientific, Waltham, MA, USA). In the second set of stainings, samples were incubated with Alexa Fluor 647–conjugated mouse anti–desmocollin-2 antibody (clone; 7G6, NOVUS Biologicals, Abingdon, UK) and Alexa Fluor 488–conjugated mouse anti-occludin antibody (clone: OC-3F10, Invitrogen, Thermo Fischer Scientific). In the third set of stainings, samples were incubated with Alexa Fluor 647–conjugated mouse anti–ZO-1 antibody (clone: ZO1-1A12, Invitrogen, Thermo Fischer Scientific) and rabbit anti–claudin-1 antibody (ab15098, Invitrogen, Thermo Fischer Scientific), together with donkey anti-rabbit Alexa Fluor 555–conjugated secondary antibody (A31572, Invitrogen, Thermo Fischer Scientific). All antibodies were diluted in washing buffer containing 0.1% BSA-C (Aurion, Immuno Gold Reagents & Accessories Wageningen, The Netherlands) and incubated for 2 h each, except for the secondary antibodies, which were incubated for 30 min. Negative controls were included for each tissue section and consisted of incubation with the secondary antibody alone, when applicable.

All tissue sections were counterstained with 4′,6-diamidino-2-phenylindole (DAPI; Molecular Probes, Life Technologies, CA, USA), washed in Milli-Q water, and thereafter mounted with Fluorescent Mounting Medium (Dako, Carpinteria, CA, USA).

Hematoxylin and eosin (H&E) staining was performed on one of the two biopsies per patient collected at 24 h PA. Briefly, tissue sections were fixed in 4% formaldehyde, stained in hematoxylin (2 min), rinsed in tap water, stained in eosin (30 s) and rinsed in tap water. Sections were then dehydrated with 70%, 95% and 100% ethanol followed by submersion in Xylene. All tissue sections were scanned into digital images using a Pannoramic MIDI II Scanner (3DHistech, Budapest, Hungary).

### Quantitative image analysis

**Figure 2:**
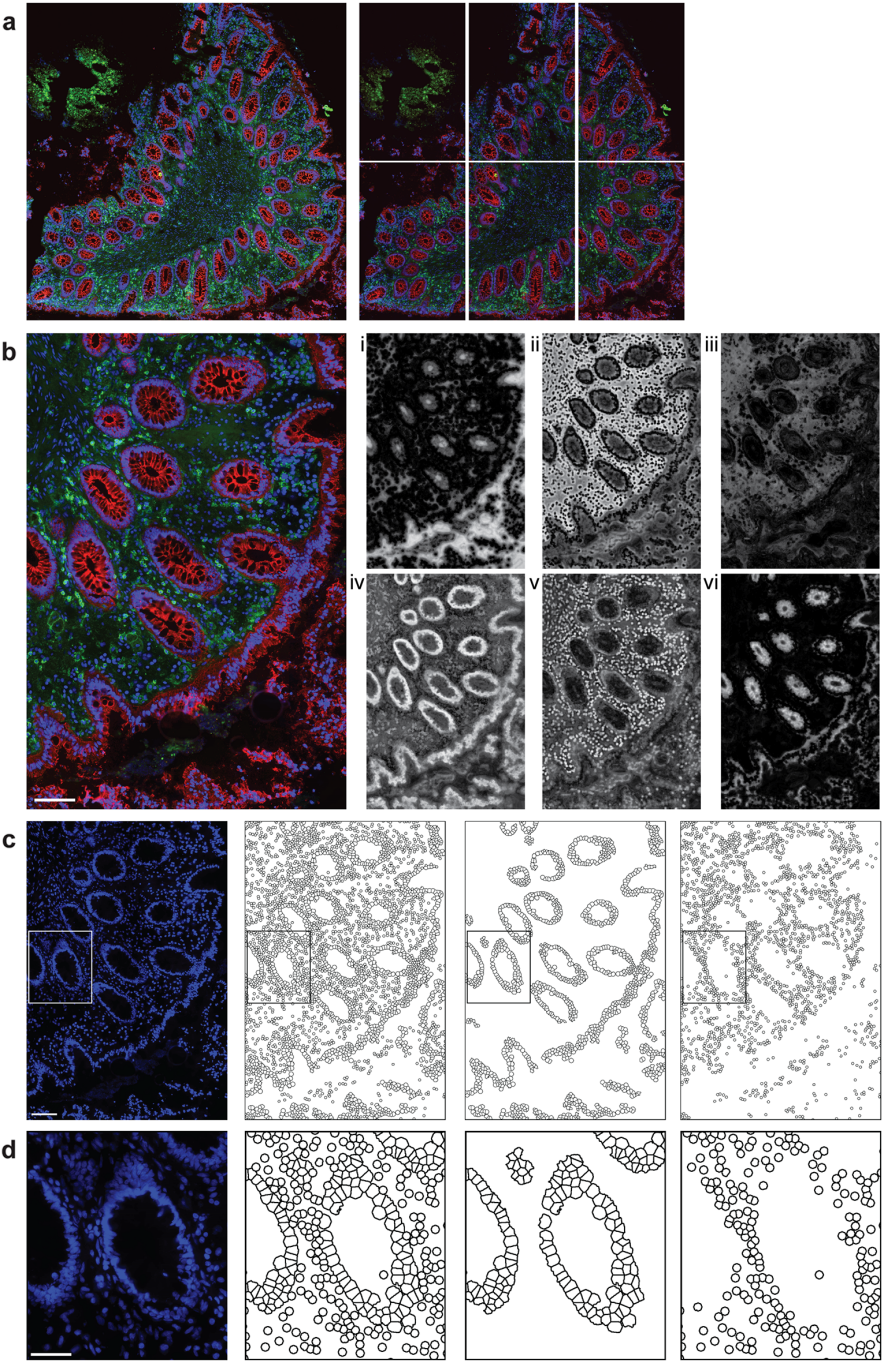
Digital image analysis workflow of the raw image processing and structure identification. (**a**) Representative raw image of human rectal tissue stained for E-cadherin (red) and CD4 (green) and counterstained for 4′,6-diamidino-2-phenylindole (DAPI; blue)(left). Raw image split into six equal-sized images in Matlab (right). (**b**) Example image of one section (left) and probability maps (right) generated from Ilastik to identify different compartments within the image: (i) black background (tissue detection), (ii) tissue background, (iii) green autofluorescence, (iv) epithelial (EP) cells, (v) lamina propria (LP) cells, (vi) epithelial junction protein. (**c**) Raw image of DAPI staining used for cell identification (left) and digitalized image (right) of all identified cells, EP cells, and LP cells, respectively. Representative images were selected based on median expression for each individual marker. Cell identification was performed in CellProfiler. (**d**) Magnified images of the outlines highlighted in (**c**). All scale bars represent 100 µm.

Scanned whole sections were exported as .tif files using Pannoramic viewer (version 1.15.4.43061), after which they were divided into six smaller segments on MATLAB (version R2016(9.0.0.341360)) (Fig. 2a). The pixel-based machine learning software Ilastik^25^ (version 1.3.3post3) was used to generate probability maps to classify cells as either epithelial (EP) or lamina propria (LP), identify epithelial tissue, and identify areas with background noise (Fig. 2b–d). The raw .tif files along with the probability maps were uploaded to CellProfiler^26^ (version 4.2.1), where EP and LP cells were enumerated, expression of the EJPs was assessed, and CD4^+^ cells were quantified (Supplementary Data and Supplementary Table S1).

**Figure 3:**
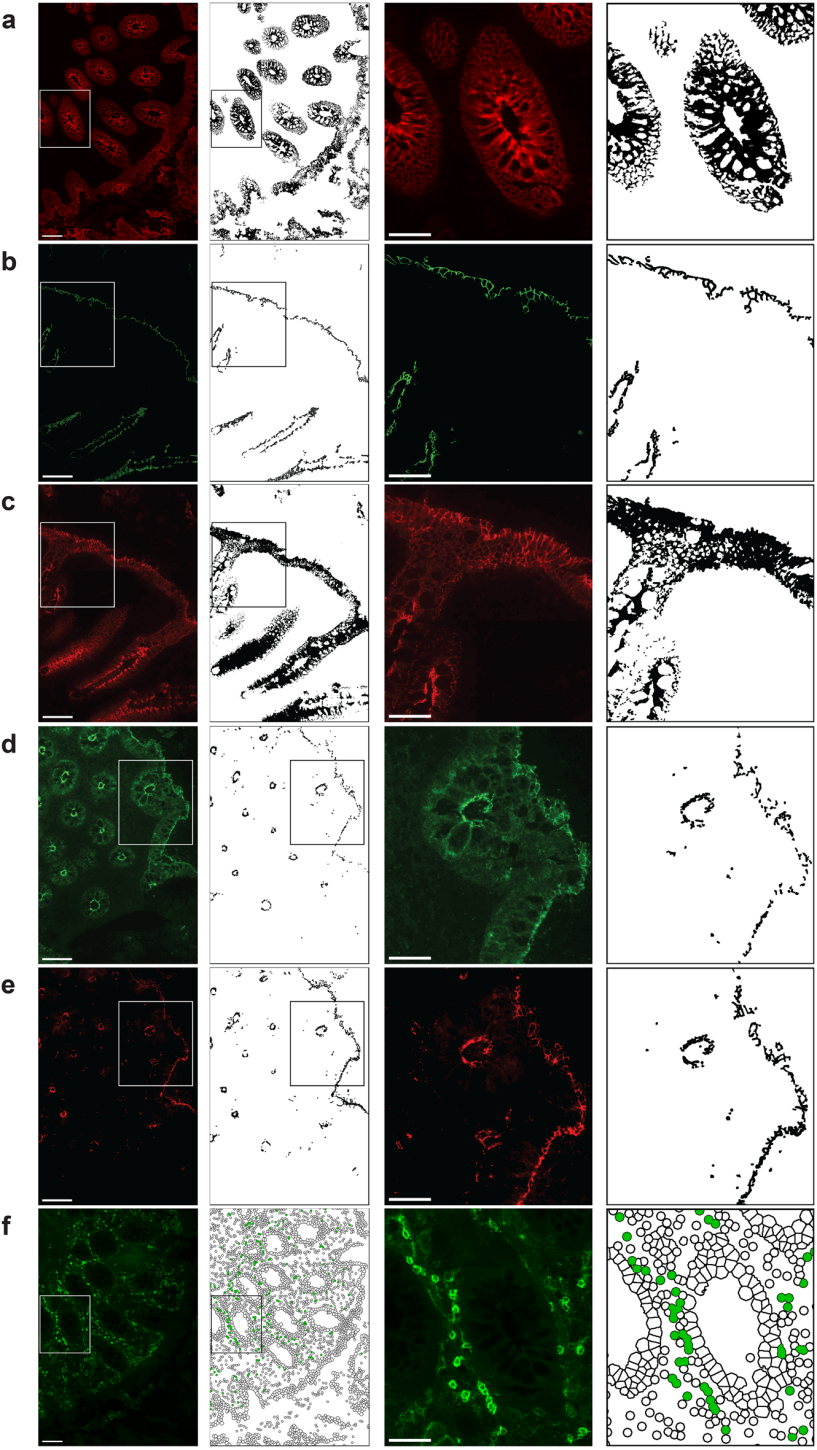
Digital bioimage analysis workflow for the analysis of epithelial junction proteins and CD4^+^ cells. (**a**–**e**) Representative raw and digitalized images of the epithelial junctional proteins (EJPs): (**a**) E-cadherin, (**b**) occludin, (**c**) desmocollin-2, (**d**) claudin-1, and (**e**) zonula occludens-1 (ZO-1). Identified EJPs are illustrated in black in the digitalized images. Epithelial tissue was identified based on probability maps generated in Ilastik. EJPs within the epithelial regions were identified in CellProfiler, and the epithelial coverage and mean fluorescence intensity (MFI) were calculated. (**f**) Representative raw and digitalized images of CD4 expression. CD4^+^ cells were quantified in CellProfiler and classified as either lamina propria (LP) or intra-epithelial (intra-EP) based on the probability maps generated in Ilastik and are illustrated in green in the digitalized images. The first column shows the raw images of each staining, the second column shows the digitalized images of each marker, the third column represents a magnified region in the raw image highlighted in the previous columns, and the fourth column shows the digitalized image of the magnified region. Scale bars represent 100 µm in the first column and 50 µm in the third column.

Briefly, cells were identified by DAPI staining and classified as either EP or LP, as defined from the epithelial probability map (Fig. 2c, d). To assess the expression of the different EJPs, only the EP tissue was of interest. EP tissue was therefore identified based on the probability maps, and each EJP was subsequently identified individually within that region (Fig. 3a–e). To quantify the levels of protein distribution, the percentage of the total assessed EP area that was stained for each of the five EJP markers (% EJP coverage) was calculated. The mean fluorescence intensity (MFI) of the EP tissue was calculated in arbitrary units (AU) as an indicator of protein expression for each marker.

Evaluation of CD4 expression was performed in both the EP and LP compartments. Cells were defined as CD4^+^ if they had an overlap of > 64 pixels with the identified CD4 staining. They were further classified as either intra-EP or LP CD4^+^ cells, depending on their spatial localization (EP or LP) (Fig. 3f). The distance of LP CD4^+^ cells from the epithelium was also calculated, and the MFI of CD4 staining was assessed as a proxy for CD4 protein expression. An adjusted bioimage analysis workflow was developed to assess potential influence of lymphoid aggregates, which contain densely packed cells, including CD4^+^ cells which may impact the CD4 data.

All workflows were developed blinded for study groups to not introduce any bias. Detailed descriptions of the bioimage analysis workflows are available at: https://github.com/MathiasFranzenBoger/Quantification-of-rectal-immune-cells-and-epithelial-junctional-proteins.

### Statistical analysis

Multiple comparison analysis was performed using the Friedman test, followed by Dunn's post-hoc test to assess differences between the selected biomarkers at the three different time points (baseline, 1 h, and 24 h). Mann–Whitney U test was used to assess differences between the two treatment groups (placebo and Q-GRFT). Differences were considered significant if the p value was < 0.05. All statistical analyses were performed using Prism 9.4.0 (GraphPad).

## Results

### Clinical characteristics and basal sample parameters

Fifteen out of the 18 study participants completed the study. Of these, 8 were cisgender males and 7 were cisgender females (Q-GRFT: 7 males and 4 females; placebo: 1 male and 3 females). The median age for all study participants was 29 years (range 18–40 years), similarly the median age for the Q-GRFT group was 29 years (range 18–40), and the placebo group was 28.5 (range 28–36).

A total of 90 rectal biopsies were collected from the 15 study participants including two biopsies obtained at each of the three timepoints. With the use of our digital bioimage analysis workflows, we objectively evaluated the in-situ immunofluorescence staining performed on tissue sections of the biopsies. This allowed us to distinguish between cells present in the EP and LP compartments (Fig. 2c and d), in addition to determining the expression patterns of the markers of interest. Both biopsies collected at each timepoint were utilized in the analysis of CD4 and E-cadherin and an average per participant and timepoint was calculated, while the dual staining for ZO-1 together with claudin-1, and desmocollin-2 together with occludin was performed on consecutive sections from one of the biopsies at each timepoint for the 15 participants per timepoint in each staining set.

General tissue characteristics were assessed for all timepoints. Combining the analysis of all markers at all timepoints revealed that the median_all timepoints_ total tissue area was 247 mm^2^, the median_all timepoints_ total number of cells was 1,165,895 and the median_all timepoints_ cell density was 4713 cells/mm^2^. The median_all timepoints_ percentage of EP cells was 45%, and the median_all timepoints_ percentage of LP cells was 55% (Table 1a). No significant differences were seen when comparing these general tissue characteristics across timepoints (BL, 1 h PA, 24 h PA) or when comparing the two treatment groups (Q-GFRT, placebo) (Table 1b).

**Table 1:** (a) Total sum of the tissue parameters assessed in all study participants combined; (b) examined tissue characteristics per biopsy (median values are given).

(a)							
Timepoint	Tissue area (mm^2^)	Total cells	Cell density (cells/mm^2^)	% EP cells	% LP cells		
BL	240.44	1,114,575	4636	45.26	54.74		
1 h PA	270.52	1,280,187	4732	42.78	53.30		
24 h PA	247.36	1,165,895	4713	46.70	57.22		

(b)							
Timepoint	Treatment		Tissue area (mm^2^)	Total cells	Cell density (cells/mm^2^)	% EP cells	% LP cells
BL	Placebo	Median	8.23	39,781	4644	45.26	54.74
		Min	4.08	19,668	4369	42.78	53.30
		Max	16.39	70,573	5276	46.70	57.22
	Q-GRFT	Median	13.53	61,504	4388	47.29	52.71
		Min	5.17	1921	3619	37.12	42.83
		Max	19.61	110,059	5616	57.17	62.88
1 h PA	Placebo	Median	11.34	48,855	4789	46.30	53.70
		Min	4.71	22,148	4124	43.48	48.36
		Max	16.72	83,117	4924	51.64	56.52
	Q-GRFT	Median	14.35	63,046	4745	45.82	54.18
		Min	6.22	26,539	4271	36.98	43.77
		Max	24.34	114,671	5275	56.23	63.02
24 h PA	Placebo	Median	14.87	69,053	4591	44.17	55.83
		Min	11.98	56,234	4407	41.39	52.37
		Max	15.78	70,886	4849	47.63	58.61
	Q-GRFT	Median	11.7	56,904	4758	43.58	56.42
		Min	5.3	25,979	4075	31.93	39.81
		Max	21.58	96,963	5205	60.19	68.07

A total of 90 rectal biopsies were collected from 15 healthy participants treated with Q-GRFT (n = 11) and the placebo (n = 4) at three different timepoints (BL, 1 h PA and 24 h PA) in a 2:1 ratio, respectively. Abbreviations: BL, baseline; PA, post application; EP, epithelial; LP, lamina propria.

### Assessment of the rectal epithelial barrier

**Figure 4:**
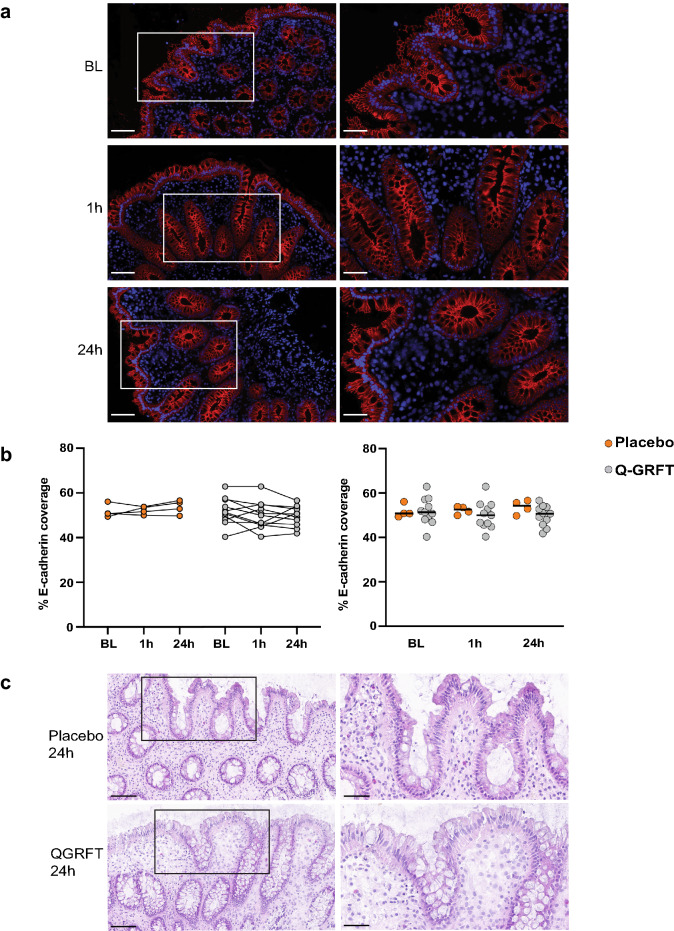
Q-GRFT treatment did not affect the epithelial E-cadherin coverage. (**a**) Representative fluorescent image showing E-cadherin (red) and 4′,6-diamidino-2-phenylindole (DAPI) nuclear staining (blue) in rectal tissue collected at the different timepoints. Images to the left show a selected tissue area at 20× magnification (scale bar = 100 µm), and the images to the right show the selected tissue (within the white rectangle) at 40× magnification (scale bar = 50 µm). (**b**) The graphs (between timepoints on the left and Q-GRFT vs. placebo on the right) show the % E-cadherin coverage (percentage of the total assessed EP area that stained positively for E-cadherin) at the different timepoints for the placebo (orange; n = 4) and Q-GRFT (grey; n = 11) groups. (**c**) Representative H&E images of placebo and Q-GRFT at 24 h PA. Images to the left show a selected tissue area at 20× magnification (scale bar = 100 µm). Images to the right demonstrates the selected tissue (within the black rectangle) at 40× magnification (scale bar = 50 µm). All data are presented as median values across patient groups. Statistical significance was determined using the Friedman test, followed by Dunn's post-hoc test, when comparing results between the three timepoints. The Mann Whitney U test was used for comparisons between the Q-GRFT and placebo groups. Representative images were selected from the median E-cadherin % coverage baseline values. Abbreviations: BL, baseline; 1 h and 24 h represent the hours after application of the rectal douche (either Q-GRFT or placebo).

**Figure 5:**
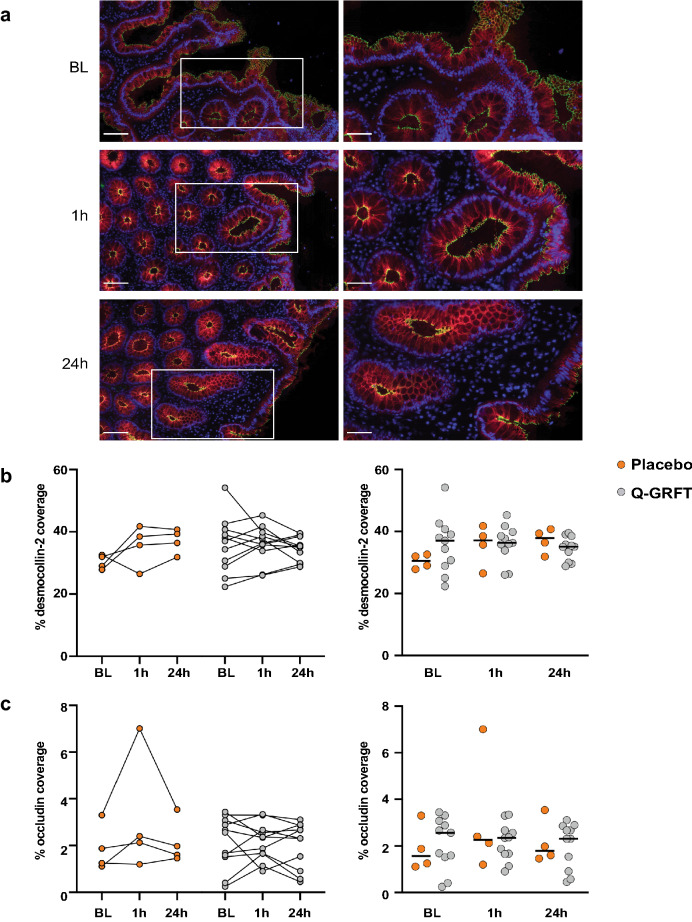
Q-GRFT treatment did not reduce epithelial coverage of desmocollin-2 and occludin. (**a**) Representative fluorescent image showing desmocolin-2 (red), occludin (green), and 4′,6-diamidino-2-phenylindole (DAPI) nuclear staining (blue) in rectal tissue collected at the different timepoints. Images to the left show a selected tissue area at 20× magnification (scale bar = 100 µm), and the images to the right show the selected tissue (within the white rectangle) at 40× magnification (scale bar = 50 µm). (**b**, **c**) The graphs (between timepoints on the left and Q-GRFT vs. placebo on the right) show the % coverage of (**b**) desmocollin-2 and (**c**) occludin (percentage of the total assessed EP area that stained positively for each of these EJP markers) at the different timepoints for the placebo (orange; n = 4) and Q-GRFT (grey; n = 11) groups. All data are presented as median values across patient groups. Statistical significance was determined using the Friedman test, followed by Dunn's post-hoc test, when comparing results between the different timepoints. The Mann Whitney U test was used for comparisons between the Q-GRFT and placebo groups. Representative images were selected from the median occludin % coverage baseline values. Abbreviations: BL, baseline; 1 h and 24 h represent the hours after application of the rectal douche (either Q-GRFT or placebo).

**Figure 6:**
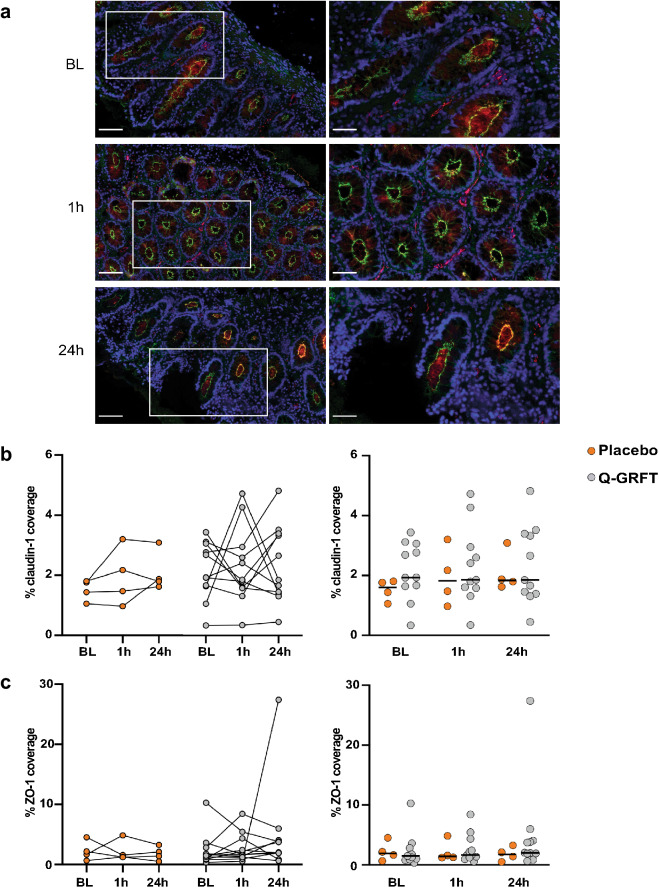
Epithelial coverage of claudin-1 and ZO-1 was unaffected by treatment with Q-GRFT. (**a**) Representative fluorescent image showing claudin-1 (green), zonula occludens-1 (ZO-1; red), and 4′,6-diamidino-2-phenylindole (DAPI) nuclear staining (blue) in rectal tissue collected at the different timepoints. Images to the left show a selected tissue area at 20× magnification (scale bar = 100 µm), and the images to the right show the selected tissue (within the white rectangle) at 40× magnification (scale bar = 50 µm). (**b**, **c**) The graphs (between timepoints on the left and Q-GRFT vs. placebo on the right) show the % coverage of (**b**) claudin-1 and (**c**) ZO-1 (percentage of the total assessed EP area that stained positively for each of these EJP markers) at the different timepoints for the placebo (orange; n = 4) and Q-GRFT (grey; n = 11) groups. All data are presented as median values across patient groups. Statistical significance was determined using the Friedman test, followed by Dunn's post-hoc test, when comparing results between the different timepoints. The Mann Whitney U test was used for comparisons between the Q-GRFT and placebo groups. Representative images were selected from the median claudin-1% coverage baseline values. Abbreviations: BL, baseline; 1 h and 24 h represent the hours after application of the rectal douche (either Q-GRFT or placebo).

Protein expression of the five selected EJPs was measured to obtain comprehensive information regarding rectal epithelial stability, integrity, plasticity, and capacity to restrict paracellular diffusion of molecules and pathogens into the epithelium. E-cadherin and desmocollin-2 surrounded the individual epithelial cells, while occludin, claudin-1, and ZO-1 were situated apically on the cells (Figs. 4a, 5a, and 6a). Hence, we assessed the percentage of the total assessed EP area that stained positively for each of the five EJP markers (% EJP coverage).

Rectal samples from the two study groups at the three different timepoints were assessed, and a median of 2 mm^2^ EP area was examined in each biopsy sample (Supplementary Table 2). E-cadherin and desmocollin-2 had clearly the largest coverage percentages, with a median % EJP coverage for all samples and timepoints of 50.9% for E-cadherin and 36.7% for desmocollin-2. These percentages were much lower for occludin (2.3%), claudin-1 (1.8%), and ZO-1 (1.8%). Neither of the five EJPs exhibited a significant difference in % EJP coverage between timepoints or between Q-GRFT and placebo groups (Figs. 4a, b, 5 and 6). Likewise, when MFI for each EJP was assessed as a proxy for protein expression levels, no significant differences were observed for any EJP when comparing timepoints or study groups (Supplementary Fig. 1).

H&E staining was performed on the biopsies collected at 24 h PA to assess general signs of epithelial disruption or leukocyte influx and visual examination revealed no such indication (Fig. 4c).

### Assessment of CD4^+^ cells

CD4^+^ T cells are the main target cells for HIV and are abundant in the rectum. The lower gastrointestinal tract has been estimated to contain more than 60% of all T cells within the human body^27^. CD4 is also expressed on macrophages and dendritic cells, which are also target cells for HIV, and all three of these CD4-expressing cell populations are present in the rectum^28^. The single-layered, simple columnar epithelium of the rectum creates a delicate barrier and is therefore prone to tearing, making this tissue increasingly susceptible for HIV acquisition^29–31^.

**Figure 7:**
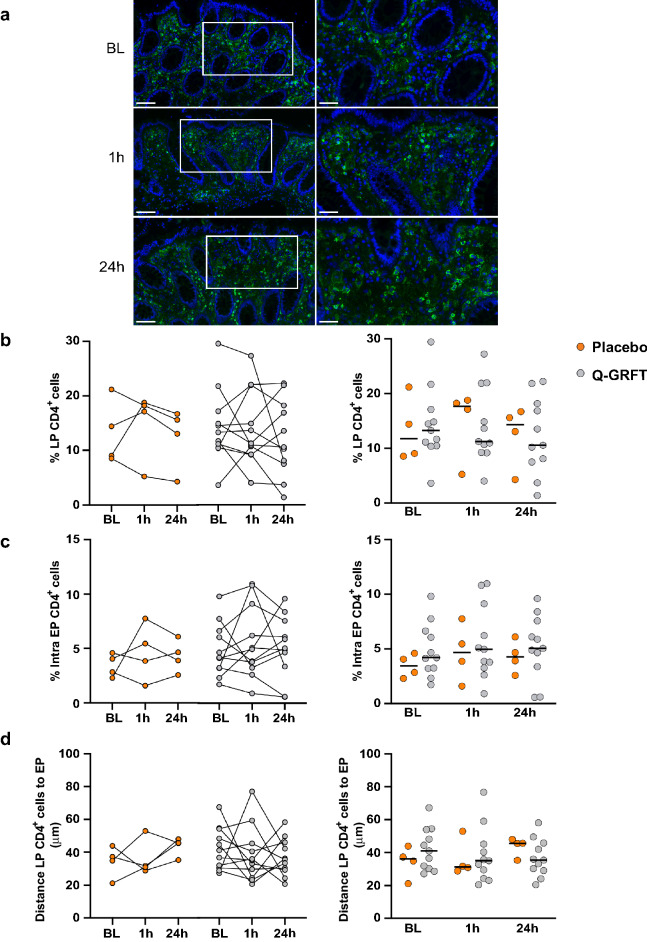
Q-GRFT treatment did not affect the magnitude or distribution of CD4^+^ cells. (**a**) Immunofluorescent images showing CD4^+^ cells (green) and 4′,6-diamidino-2-phenylindole (DAPI) nuclear staining (blue). Images to the left show a selected tissue area at 20× magnification (scale bar = 100 µm), and the images to the right show the selected tissue (within the white rectangle) at 40× magnification (scale bar = 50 µm). Representative images were selected based on median percentage of CD4^+^ LP cells. Graphs displaying the median percentage of CD4^+^ cells in the two study groups within (**b**) the LP compartment and (**c**) the EP compartment. (**d**) The graphs show the average distance from the epithelial barrier to the CD4^+^ cells in the LP in each study group. All graphs show both the grouped analysis (right) and comparison of the different timepoints for the placebo (orange; n = 4) and Q-GRFT (grey; n = 11) (left). All data are presented as the median values from the participant data. Statistical significance was determined using the Friedman test, followed by Dunn's post-hoc test, when comparing results between the different timepoints. The Mann Whitney U test was used for comparisons between the Q-GRFT and placebo groups. Abbreviations: LP, lamina propria; BL, baseline; 1 h and 24 h represent the hours after application of the rectal douche (either Q-GRFT or placebo).

The majority of CD4^+^ cells present in the rectal mucosa were located in the LP (Fig. 7a and Supplementary Table 3). There was a median of 1583 CD4^+^ cells per tissue section across all timepoints, with a median of 341 intra-EP CD4^+^ cells and 1247 LP CD4^+^ cells. A significant difference was observed when comparing the intra-EP CD4^+^ cell count at 1 h PA between patients treated with Q-GRFT and those treated with placebo (median intra-EP CD4^+^ cells: 576 cells vs. 281 cells, p = 0.02). However, no other significant differences in intra-EP CD4^+^ or LP CD4^+^ cell counts were observed when compared across timepoints or between treatment groups (Supplementary Table 3). No significant differences were observed in the percentage of CD4^+^ LP cells or intra-EP CD4^+^ cells (of the total LP and EP cells, respectively) at the different timepoints or between the Q-GRFT and placebo groups (Fig. 7b and c). Similarly, no differences could be observed when comparing the CD4^+^ cell density (number of CD4^+^ cells/mm^2^) in either compartment (Supplementary Fig. 2).

Additionally, the MFI of the CD4^+^ cells revealed no significant differences when compared across different timepoints or between the two groups (Supplementary Fig. 3).

We also assessed the distance from the epithelial border to CD4^+^ cells present in the LP as a theoretical measure of the distance required for an HIV particle to travel to reach its target cells. CD4^+^ cells were located a mean of 36 µm (range, 21–77 µm) from the epithelial border. No significant differences were observed in the distance of CD4^+^ cells from the epithelial border across all timepoints or when comparing Q-GRFT and placebo groups (Fig. 7d).

Lymphoid aggregates are dispersed throughout the lower gastrointestinal tract including the rectal compartment. Cells within these aggregates are densely clustered which could potentially skew the data. To correct for these, an adjusted version of the bioimage analysis workflow was created where lymphoid aggregates were manually removed from subsequent analysis. Using this workflow, no significant differences could be observed in either the percentage of CD4^+^ LP cells, intra-EP CD4^+^ cells or the distance from the CD4^+^ LP cells to the epithelial border when comparing across timepoints or between Q-GRFT and placebo (Supplementary Fig. 4).

Collectively, our data indicated that Q-GRFT administered via a rectal douche dosage form did not affect the distribution or expression levels of CD4^+^ cells or of any of the five selected EJPs.

## Discussion

In this study, we demonstrated that topical application of rectal douche containing Q-GRFT, a promising anti-HIV agent, did not alter the expression or distribution of potential biomarkers of HIV susceptibility in the rectal mucosa of healthy volunteers participating in a phase 1 clinical trial. RAI has been considered a high-risk activity for HIV transmission because of the fragile single-cell epithelial layer of the rectal mucosa, which is in immediate proximity to the vascular submucosa. In addition to its structure, the abundant HIV target cells (ie, CD4^+^ cells) in the anorectal mucosa increase the susceptibility to HIV infection. Thus, it is of great importance to ensure that topical application of anti-viral agents do not alter the expression of barrier proteins and/or the presence of immune cells, especially when treatment may be prescribed for individuals with an HIV-seropositive sexual partner.

Here, we assessed six biomarkers, including a panel of five EJPs, as well as CD4^+^ cells. These CD4^+^ cells served as a marker for HIV target cells, as well as for inflammation. The EJPs were selected to represent adherens junctions (E-cadherin), desmosomes (desmocollin-2), two tight junction proteins (occludin and claudin-1), and the tight junction anchoring protein (ZO-1). Topical application of Q-GRFT did not affect the coverage or the MFI for any of the five selected EJPs, when compared across the three timepoints or when compared between the Q-GRFT and placebo groups.

A significant increase in the Q-GRFT group compared to the placebo was found in the intra-EP CD4^+^ cell at 1 h after application, but this significant difference was lost when the data were normalized to total EP cells. Hence, no significant differences were seen in the percentage of intra-EP CD4^+^ cells nor in the percentage of LP CD4^+^ cells in any of the comparisons (across timepoints or between groups). Similarly, no significant differences could be observed when assessing CD4^+^ cell density in either compartment.

Lymphoid aggregates that contain densely clustered cells, including CD4^+^ cells are dispersed throughout the lower gastrointestinal tract, including the rectal compartment. Application of the Q-GRFT rectal douche is unlikely to have caused the formation of these aggregates. However, the presence of which could influence the CD4 evaluation. Therefore, an adjusted version of the CD4 bioimaging workflow was developed. This version allowed for manual annotation of the lymphoid aggregates that were subsequently removed from the analysis. While the total CD4^+^ cell count decreased following the removal of the lymphoid aggregates, no significant differences were seen when comparing the percentage of CD4^+^ cells in either compartment across the different timepoints or when comparing the Q-GRFT and placebo groups.

Visual examination of H&E staining at 24 h after application did not reveal any indication of epithelial disruption or signs of leukocyte infiltration. Unfortunately, we did not have access to rectal lavage samples to assess soluble inflammation markers which would have been informative with regards to possible inflammatory activity.

Our data thus collectively indicate that Q-GRFT treatment did not affect the rectal epithelial barrier or the distribution and expression intensity of the CD4^+^ cells present therein. These data are in line with the results of our previous study in which we performed a thorough analysis of the effects of Q-GRFT on the rectal mucosal environment in a non-human primate model. In that pre-clinical explorative safety evaluation, we showed that neither placebo nor product induced any serious perturbations in the rectal mucosa, as assessed by proteome, microbiome, and distribution of CD4^+^ cells and E-cadherin expression^21,32^.

For a biopharmaceutical candidate for HIV prevention to be efficient, it must cover and adhere to the mucosal surface. In an ex vivo permeability study, conducted using ectocervical tissues, we showed that GRFT adhered to the superficial epithelial layer of the ectocervical epithelium^33^. Since GRFT and Q-GRFT are protein compounds with molecular weight of about 26 kDa (dimer form) they are not expected to permeate through tissues and mammalian cells and no anti-drug antibodies have been detected^20,34^. Leyva et al.^35^ have shown that a saline based enema product named Normosal-R, which was formulated in a similar base as the Q-GRFT product and the same volume (of 125 mL) was given, was distributed fully throughout the rectum, sigmoid, and descending colon. While the colorectal distribution of the Q-GRFT douche product was not directly measured in this study, a similar colorectal distribution could possibly be expected as seen with the Normosal-R enema. Collectively, these results are encouraging with regards to the use of GRFT for the prevention of not only HIV infection but also other STIs that could take advantage of a disrupted epithelium, including HSV-2 and HCV, since GRFT has also been shown to protect against these viruses in experimental studies and small animal models^14,36,37^.

HIV infection prevention strategies adjusted to the user’s risk factors, preferences, and health care availability now include several products for use as PrEP therapeutics. In addition to the well-documented effects of daily oral antiretroviral PrEP use, the use of long-acting PrEP medications or behaviorally congruent on-demand applications such as rectal douching are promising. Furthermore, clinical studies, in addition to the Q-GRFT trial, are ongoing to determine the safety, acceptability, and effectiveness of rectal anti-viral agents for preventing HIV infection through anal sex^38^. Identifying compounds that can complement oral or injectable PrEP substances is a high global health priority. However, several clinical trials of HIV infection prevention in the female genital tract reported unfavorable outcomes despite safety clearance from pre-clinical toxicity tests. Possible molecular mechanisms that may have contributed to the failures included disruption of the epithelial barrier and induction of a local inflammatory response^39–43^. The failed trials used standard small animal models and ex vivo tissue models for toxicity evaluations. These models assessed leukocyte influx and other signs of inflammation but were not specifically designed to measure additional factors of importance for HIV entry and replication, such as CD4^+^ T cell populations or shifts to a more pathogenic microbiome. There is thus an urgent need to define critical markers beyond current standard toxicity models for evaluating safety in clinical trials. We suggest that robust expression of EJPs and an unchanged number of HIV target cells in the rectal mucosa are critical toxicity markers that can complement standard safety assessments in clinical trials.

The bioimage analysis workflows used here was developed to perform high-throughput objective analysis of the rectal mucosa. Our workflows allow for large-scale quantitative image analysis, while retaining spatial information regarding the distribution of EJPs (% EJP coverage) and localization of the two CD4^+^ cell populations (intra-EP and LP CD4^+^ cells). The workflows also include the possibility to distinguish between EP, LP and intra follicular cells, which might be of interest for studies in naïve animals and for long term studies in both animals and humans.

Spatial information of tissue structures and cells adds another layer of valuable information and thus increases our understanding of how different compounds affect the mucosal borders and the cells located therein. Our workflows can easily be expanded to assess other cell types and mucosal tissues with similar epithelial morphology.

In conclusion, rectal administration of Q-GRFT did not influence the frequency, expression, and/or localization of any of the biomarkers examined at the histological level, thus suggesting its viability to be advanced as a potential HIV prevention product candidate. The work could also have implications in other therapeutic indices being explored for Q-GRFT including SARS-CoV-2 infection^17,18,44,45^.

## Supplementary Information

Supplementary Information 1.

Supplementary Figure 1.

Supplementary Figure 2.

Supplementary Figure 3.

Supplementary Figure 4.

Supplementary Table 1.

Supplementary Table 2.

Supplementary Table 3.

## Data Availability

All data generated or analysed during this study are included in this published article and its Supplementary Information files. The bioimage analysis workflow scripts and example images are available at GitHub (https://github.com/MathiasFranzenBoger/Quantification-of-rectal-immune-cells-and-epithelial-junctional-proteins).
