# Supplementary material for: A topical rectal douche product containing Q-Griffithsin does not disrupt the epithelial border or alter CD4+ cell distribution in the human rectal mucosa
Source: Sci Rep. 2023 May 9;13:7547. doi: 10.1038/s41598-023-34107-w (PMC10169179; doi:10.1038/s41598-023-34107-w)
Supplement: Supplementary file 1 — Supplementary Information 1. [file 41598_2023_34107_MOESM1_ESM.pdf]

Supplementary Data

Quantitative bioimage analysis

Scanned whole sections were exported as .tif files using Pannoramic viewer (version 1.15.4.43061) and were split into 6 smaller segments using MATLAB (version R2016(9.0.0.341360)). The resulting images were downsized to 5 % of their original size and uploaded to Ilastik[1] (version 1.3.3post3) to generate probability maps used for identification of markers of interest. The generated probability maps comprised of: **i**) black background (tissue detection), **ii**) tissue background, **iii**) green autofluorescence, **iv**) epithelial (EP) cells, **v**) lamina propria (LP) cells, **vi**) epithelial junction protein (EJP) (Fig. 2b). The split raw images were also downsized to 50% of their original size and were along with the probability maps uploaded to CellProfiler[2] (version 4.2.1) for quantitative bioimage analysis of the following.

1. Identification of epithelial- (EP) and lamina propria (LP) cells
2. Assessment of epithelial junctional proteins (EJP)
3. Classification and enumeration of $CD4^+$ cells.

Both workflows in Ilastik and CellProfiler were evaluated blinded with a training set consisting of 98 images (49 stained and 49 negative controls). The Ilastik workflow was developed by a combination of both the stained images and negative controls, while the CellProfiler workflow was developed based on the stained images and then evaluated using the negative controls and the settings were determined based on visual examination.

-Identification of epithelial (EP) and lamina propria (LP) cells

Initially, the tissue within each image was identified based on the black background probability map and laid a foundation for all further analysis. Furthermore, areas containing green autofluorescence was removed from analysis. The nucleic staining was used to classify cells in a multistep process. Initially, nuclei were identified and shrunken to a point. They were thereafter expanded by 12 pixels to acquire a more uniform nucleic classification. The identified nuclei were further classified as either EP or LP based on their overlap with the EP cell probability map. LP nuclei were expanded by 3 pixels and further classified as LP cells while EP nuclei were expanded by 15 pixels and classified as EP cells to better represent the size difference between the cell types **(Fig. 2)**. All cells within 2 pixels from the identified green autofluorescence were removed from subsequent analysis. Both cell types were thereafter enumerated, and the percentage of each cellular compartment from the total cell population was calculated.

-Assessment of epithelial junction proteins (EJPs)

To perform analysis of the epithelial junction proteins (EJP; E-cadherin, occludin, desmocollin-2, claudin-1, and ZO-1) only the epithelial compartment was of interest. The epithelial compartment was identified by excluding tissue regions identified by the probability maps for green autofluorescence, tissue background and LP cells from analysis. Thus, leaving only the epithelial tissue. The resulting images were split into greyscale images and each marker was subsequently identified and analysed. All EJPs were identified using a three-class Otsu threshold with varying middle-class classifications, pre-processing, corrections factors, and minimum intensity threshold (**Fig. 3a-e and Supplementary Table 1**).

Using the above steps, the area occupied by the epithelial tissue was assessed, followed by the area occupied by each individual marker to assess the protein distribution. A percentage was calculated comparing the area occupied by each EJP with the total area of the epithelial tissue (% EJP coverage). Furthermore, mean fluorescence intensity (MFI) was calculated for each EJP within the epithelial tissue as an indication of protein expression. The intensity of each image was rescaled to 0-1 where 1 was the maximum possible intensity.

-Classification and enumeration of CD4$^+$ cells

CD4$^+$ cells were classified in the following two-step procedure (**Fig. 3f and Supplementary Table 1**).

- To reduce autofluorescence background and artefacts, a gaussian filter (size=150 pixels) was applied and subsequently subtracted from the original image. The resulting image was then analysed using a three class Otsu threshold with the middle class classified as background and a correction factor of 1.7 followed by a minimum intensity threshold of 0.04.
- All cells containing >64 positive pixels from the segmented CD4 staining were classified as either LP or intra-EP CD4$^+$ cells depending on previous classification.

CD4$^+$ cells were enumerated, and the percentage and density of CD4$^+$ cells within the total cell population, EP cell population and LP cell population was calculated. Furthermore, lymphoid aggregates were present in some biopsies which may skew the data. An adjusted version of the workflow was therefore created. The lymphoid aggregates were manually annotated and classified as a follicular compartment. All cells within, or touching the annotation were

classified as follicular cells and removed from subsequent analysis. MFI was measured as an indication of CD4 expression.

The average distance of LP $CD4^+$ cells to the epithelial compartment, was calculated to assess availability of these cells for a theoretical virion. An Euclidian distance transformed image of the identified epithelial cell compartment was generated. Mean and upper quartile intensity (i.e., distance) of LP $CD4^+$ cells within the Euclidian distance transformed image was calculated.

The bioimage analysis workflows are available at:
https://github.com/MathiasFranzenBoger/Quantification-of-rectal-immune-cells-and-epithelial-junctional-proteins

References

1. Berg, S. *et al.* ilastik: interactive machine learning for (bio)image analysis. *Nat. Methods 2019 1612* **16**, 1226–1232 (2019).
2. Stirling, D. R. *et al.* CellProfiler 4: improvements in speed, utility and usability. *BMC Bioinformatics* **22**, (2021).
