# Supplementary material for: A topical rectal douche product containing Q-Griffithsin does not disrupt the epithelial border or alter CD4+ cell distribution in the human rectal mucosa
Source: Sci Rep. 2023 May 9;13:7547. doi: 10.1038/s41598-023-34107-w (PMC10169179; doi:10.1038/s41598-023-34107-w)
Supplement: Supplementary file 2 — Supplementary Figure 1. [file 41598_2023_34107_MOESM2_ESM.pdf]

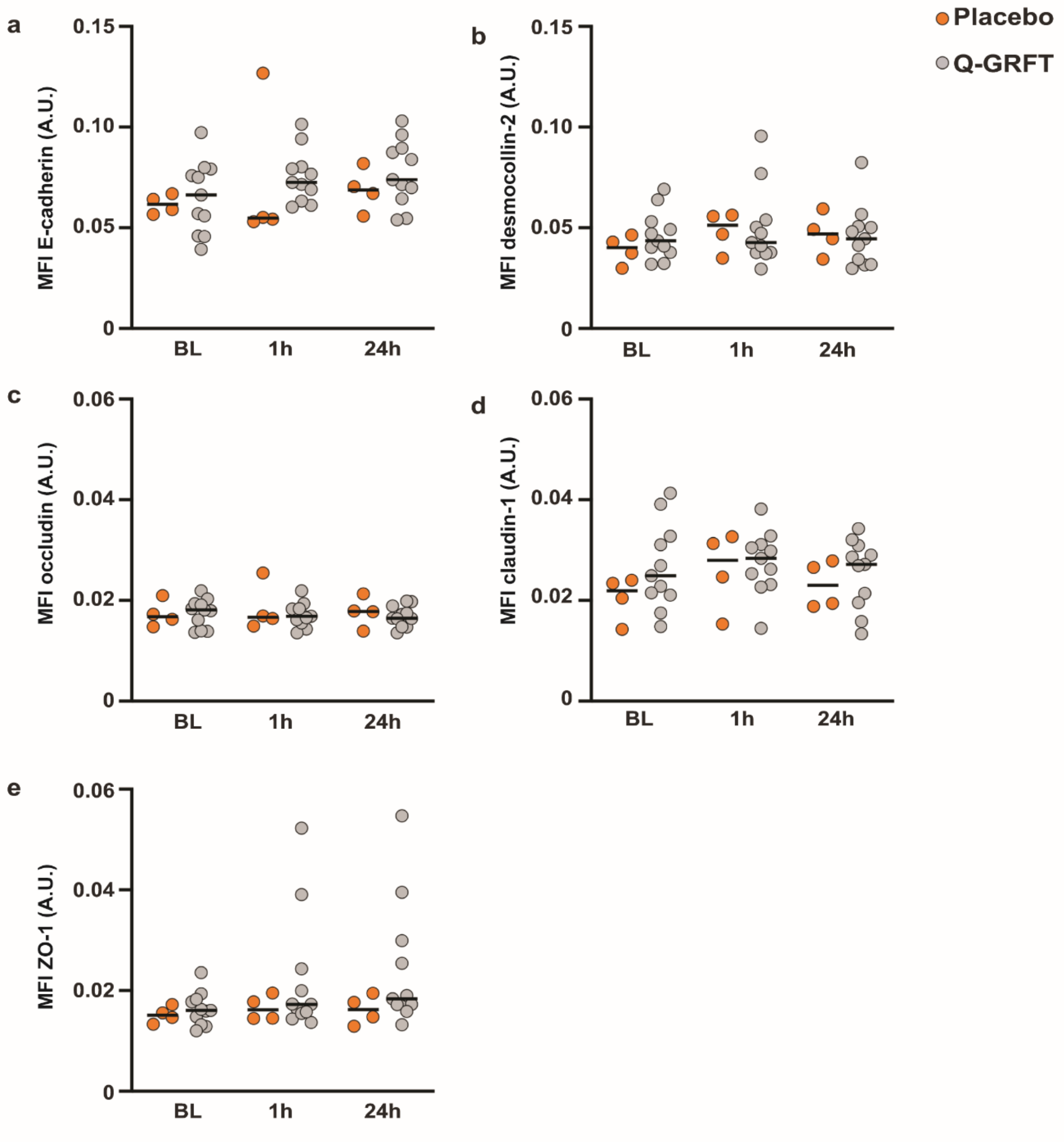

Supplementary Figure 1. Unaltered expression of EJP expression after topical treatment with Q-GRFT

The mean fluorescence intensity (MFI) of each EJP within the EP tissue was calculated in arbitrary units (AU). The graphs present the expression intensity of [a] E-cadherin, [b] desmocollin-2, [c] occludin, [d] claudin-1 and [d] ZO-1 in the Q-GRFT (grey; n=11) and placebo (orange; n=4) study groups. Statistical significance was determined using the Friedman test, followed by Dunn's post-hoc test when comparing results between the different timepoints and a Mann Whitney test performed for comparisons between the treatment groups Q-GRFT and placebo. Abbreviations: EJP, Epithelial junction protein; EP, Epithelium; BL, baseline 1h and 24h represent the hours after application of the rectal douche (either Q-GRFT or placebo).
