# Supplementary material for: A topical rectal douche product containing Q-Griffithsin does not disrupt the epithelial border or alter CD4+ cell distribution in the human rectal mucosa
Source: Sci Rep. 2023 May 9;13:7547. doi: 10.1038/s41598-023-34107-w (PMC10169179; doi:10.1038/s41598-023-34107-w)
Supplement: Supplementary file 3 — Supplementary Figure 2. [file 41598_2023_34107_MOESM3_ESM.pdf]

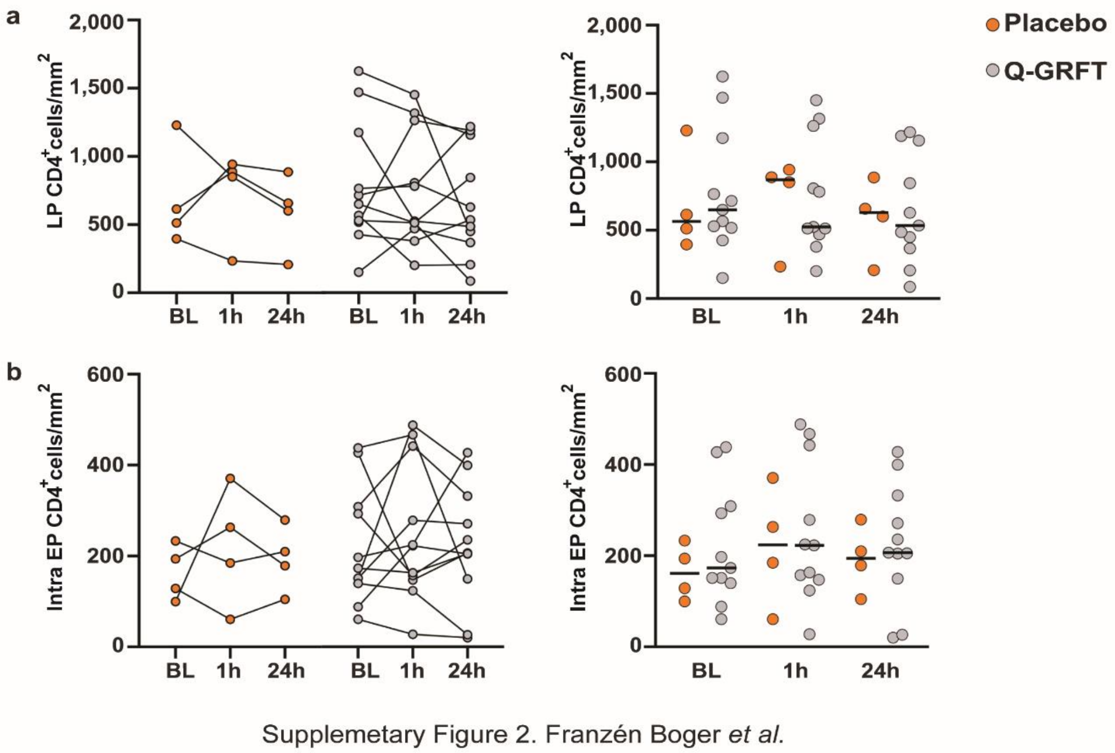

Supplementary Figure 2. Q-GRFT treatment did not affect CD4$^+$ cell density in either the lamina propria or epithelial compartment

Graphs displaying the median density of CD4$^+$ cells in the two study groups within [a] the lamina propria compartment and, [b] epithelial compartment. All graphs show both the grouped analysis (right) and comparison of the different timepoints for placebo (orange; n=4) and Q-GRFT (grey; n=11) (left). All data is presented as the median values from each participant data. Statistical significance was determined using the Friedman test, followed by Dunn's post-hoc test, when comparing results between different timepoints. The Mann Whitney U test was used for comparison between the Q-GRFT and placebo groups. Abbreviations: BL, baseline; 1h and 24 h represent the hours after application of the rectal douche (either Q-GRFT or placebo).
