# Supplementary material for: A topical rectal douche product containing Q-Griffithsin does not disrupt the epithelial border or alter CD4+ cell distribution in the human rectal mucosa
Source: Sci Rep. 2023 May 9;13:7547. doi: 10.1038/s41598-023-34107-w (PMC10169179; doi:10.1038/s41598-023-34107-w)
Supplement: Supplementary file 4 — Supplementary Figure 3. [file 41598_2023_34107_MOESM4_ESM.pdf]

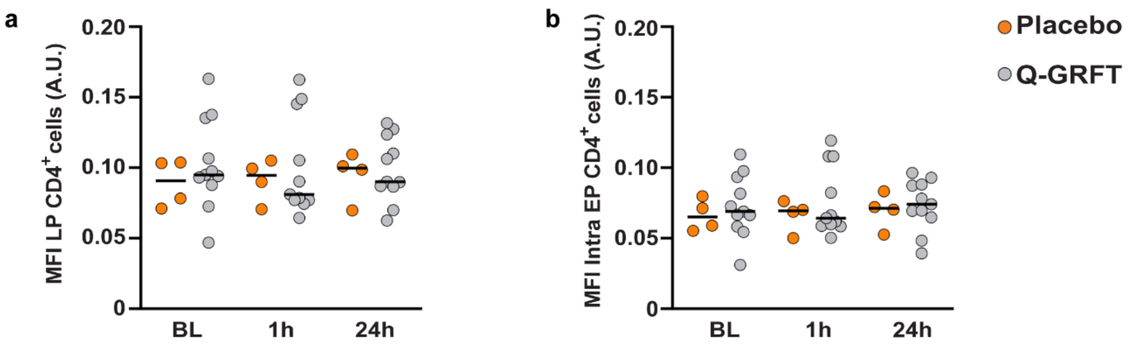

Supplemetary Figure 3. Franzén Boger *et al.*

Supplementary Figure 3. Treatment with Q-GRFT enema had no effect on the CD4 expression

The mean fluorescence intensity (MFI) of the CD4 staining was calculated in arbitrary units (AU). The graphs present the expression intensity on [a] $CD4^+$ LP- and [b] $CD4^+$ intra-EP cells at the different timepoints for the placebo (orange; n=4) and Q-GRFT (grey; n=11) study groups. Statistical significance was determined using the Friedman test, followed by Dunn's post-hoc test when comparing results between the different timepoints and a Mann Whitney test performed for comparisons between the treatment groups Q-GRFT and placebo. Abbreviations: EP, Epithelium; BL, baseline 1h and 24h represent the hours after application of the rectal douche (either Q-GRFT or placebo)
