# Supplementary material for: A topical rectal douche product containing Q-Griffithsin does not disrupt the epithelial border or alter CD4+ cell distribution in the human rectal mucosa
Source: Sci Rep. 2023 May 9;13:7547. doi: 10.1038/s41598-023-34107-w (PMC10169179; doi:10.1038/s41598-023-34107-w)
Supplement: Supplementary file 5 — Supplementary Figure 4. [file 41598_2023_34107_MOESM5_ESM.pdf]

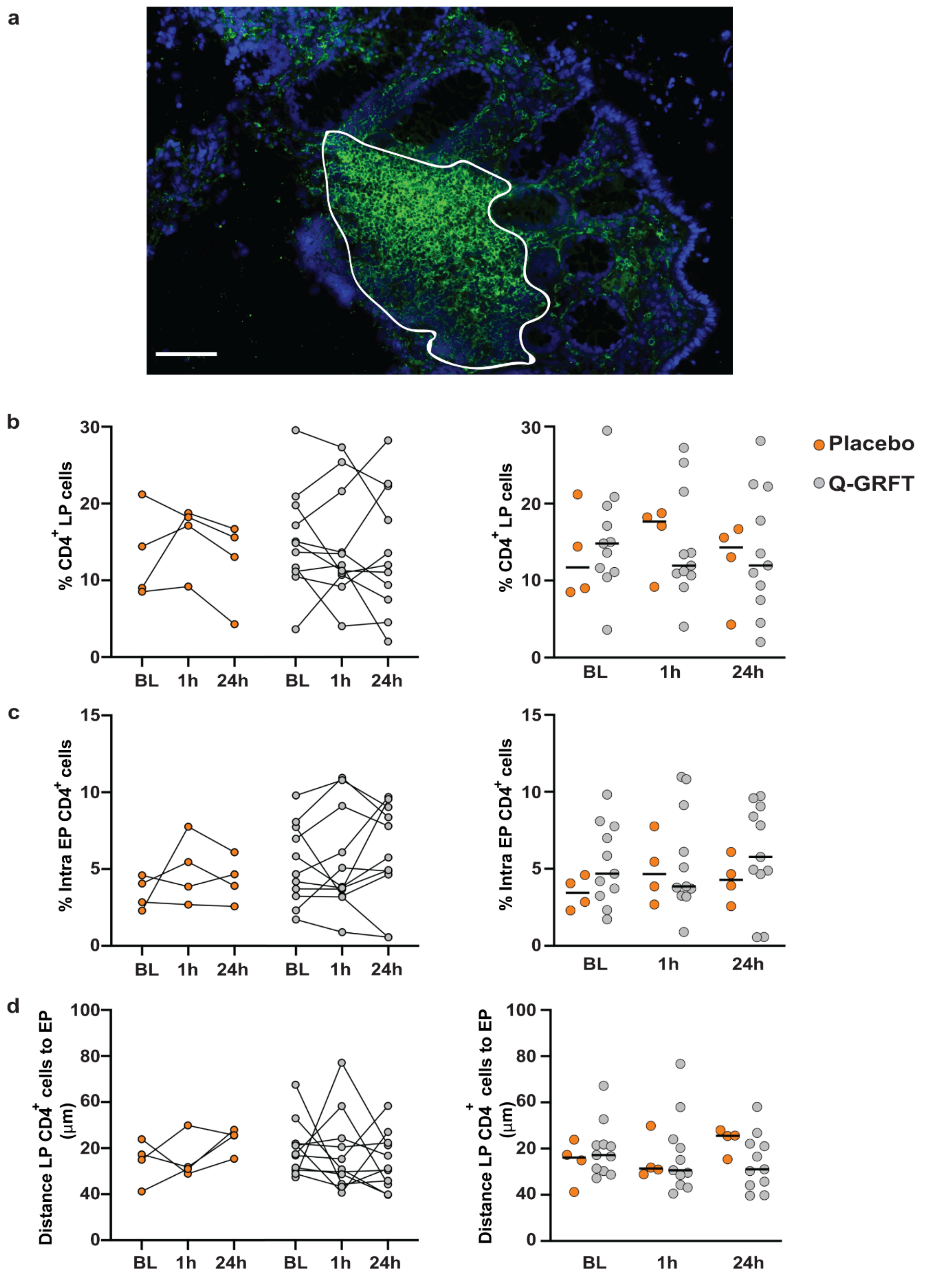

Supplemetary Figure 4. Franzén Boger *et al.*

Supplementary Figure 4. Q-GRFT treatment did not affect the magnitude or distribution of CD4$^+$ cells following removal of lymphoid aggregates.

[a] Immunofluorescent image showing CD4$^+$ cells (green) and 4´,6-diamidino-2-phenylindole (DAPI) nuclear staining (blue). The white line demonstrates the manual annotation for lymphoid aggregate removal (scale bar=100 μm). Graphs displaying the median percentage of CD4$^+$ cells following removal of lymphoid aggregates in the two study groups within [b] the lamina propria compartment and, [c] epithelial compartment. [d] The graphs show the average distance between epithelial compartment to the CD4$^+$ lamina propria cells in each study group. All graphs show both the grouped analysis (right) and comparison of the different timepoints for the placebo (orange; n=4) and Q-GRFT (grey; n=11) (left). All data is presented as the median values from each participant data. Statistical significance was determined using the Friedman test, followed by Dunn's post-hoc test, when comparing results between different timepoints. The Mann Whitney U test was used for comparison between the Q-GRFT and placebo groups. Abbreviations: BL, baseline; 1h and 24 h represent the hours after application of the rectal douche (either Q-GRFT or placebo)
