# Supplementary material for: A topical rectal douche product containing Q-Griffithsin does not disrupt the epithelial border or alter CD4+ cell distribution in the human rectal mucosa
Source: Sci Rep. 2023 May 9;13:7547. doi: 10.1038/s41598-023-34107-w (PMC10169179; doi:10.1038/s41598-023-34107-w)
Supplement: Supplementary file 6 — Supplementary Table 1. [file 41598_2023_34107_MOESM6_ESM.pdf]

Supplementary Table 1.
Settings for the quantitative bioimage analysis workflows for each individual marker

Protein (Channel)	Clone	Exposure (ms)	Filtering (pixels)	Thresholding method	Correction factor	Lower threshold (0-1)	Object size parameter
**Set 1: CD4 (Cy3)**	EPR6855	200	Subtract Gaussian blur (size=150) Gaussian blur (size=1)	Three-class Otsu Middle class background	1.7	0.04	12 - ∞
**Set 1: E-cadherin (Cy5)**	36	100	NA	Three-class Otsu Middle class foreground	0.8	0.03	4 - ∞
**Set 2: occludin (FITC)**	OC-3F10	150	Enhance neurites (size=8) Gaussian blur (size=1)	Three-class Otsu Middle class foreground	1.0	0.04	6 - ∞
**Set 2: desmocollin-2 (Cy5)**	7G6	200	Gaussian blur (size=1)	Three-class Otsu Middle class foreground	1.0	0.03	4 - ∞
**Set 3: claudin-1 (Cy3)**	ab15098	100	Enhance neurites (size=8) Gaussian blur (size=1)	Three-class Otsu Middle class background	0.7	0.05	6 - ∞
**Set 3: ZO-1 (Cy5)**	ZO1-1A12	100	Enhance neurites (size=8) Gaussian blur (size=1)	Three-class Otsu Middle class background	0.6	0.04	6 - ∞
