# Supplementary material for: A topical rectal douche product containing Q-Griffithsin does not disrupt the epithelial border or alter CD4+ cell distribution in the human rectal mucosa
Source: Sci Rep. 2023 May 9;13:7547. doi: 10.1038/s41598-023-34107-w (PMC10169179; doi:10.1038/s41598-023-34107-w)
Supplement: Supplementary file 7 — Supplementary Table 2. [file 41598_2023_34107_MOESM7_ESM.pdf]

Supplementary Table 2.
Total epithelial area assessed for each of the three staining sets performed

Time-point	Treatment		EP tissue area (mm^2) [E-cadherin/CD4]	EP tissue area (mm^2) [occludin/desmocollin-2]	EP tissue area (mm^2) [ZO-1/Claudin-1]
**BL**	**Placebo**	Median	1.43	1.20	1.07
		Min	0.8	0.45	0.47
		Max	2.24	2.76	2.75
	**Q-GRFT**	Median	2.54	1.94	1.76
		Min	0.31	0.74	0.62
		Max	4.16	3.56	3.33
**1 h PA**	**Placebo**	Median	1.36	1.55	1.99
		Min	0.88	0.86	0.82
		Max	2.82	3.6	3.03
	**Q-GRFT**	Median	2.5	2.09	2.21
		Min	1.3	1.14	0.65
		Max	4.11	5.46	5.75
**24 h PA**	**Placebo**	Median	2.44	2.47	2.03
		Min	1.8	1.66	1.61
		Max	2.65	2.71	2.49
	**Q-GRFT**	Median	2.13	1.83	2.02
		Min	0.82	0.78	0.94
		Max	4.34	2.73	3.72

Staining Set 1: E-cadherin & CD4; Staining Set 2: occludin & desmocollin-2; Staining Set 3: ZO-1 & claudin-1. Data is separated into each study group; Q-GRFT (n=11) and placebo (n=4) and represented as values per biopsy. Abbreviations: BL, baseline; PA, post application; EP, epithelial; LP, lamina propria.
