# Supplementary material for: A topical rectal douche product containing Q-Griffithsin does not disrupt the epithelial border or alter CD4+ cell distribution in the human rectal mucosa
Source: Sci Rep. 2023 May 9;13:7547. doi: 10.1038/s41598-023-34107-w (PMC10169179; doi:10.1038/s41598-023-34107-w)
Supplement: Supplementary file 8 — Supplementary Table 3. [file 41598_2023_34107_MOESM8_ESM.pdf]

**Supplementary Table 3**.
***Number of CD4⁺cells within the rectal compartment***

| Timepoint | Treatment | | No. of total CD4⁺ cells | No. of CD4⁺ intra-EP cells | No of CD4⁺ LP cells |
|---|---|---|---|---|---|
| **BL** | **Placebo** | Median | 1, 316 | 279 | 1, 037 |
| | | Min | 432 | 80 | 352 |
| | | Max | 1, 763 | 331 | 1, 433 |
| | **Q-GRFT** | Median | 2, 277 | 527 | 1, 688 |
| | | Min | 414 | 57 | 280 |
| | | Max | 4, 445 | 820 | 3, 939 |
| **1 h PA** | **Placebo** | Median | 985 | 281* | 743 |
| | | Min | 879 | 172 | 629 |
| | | Max | 2, 110 | 338 | 1, 772 |
| | **Q-GRFT** | Median | 1, 849 | 576* | 1, 456 |
| | | Min | 498 | 49 | 449 |
| | | Max | 5, 617 | 1, 752 | 3, 865 |
| **24 h PA** | **Placebo** | Median | 1, 866 | 397 | 1, 469 |
| | | Min | 945 | 277 | 668 |
| | | Max | 2, 982 | 712 | 2, 270 |
| | **Q-GRFT** | Median | 1, 255 | 284 | 1, 010 |
| | | Min | 315 | 57 | 258 |
| | | Max | 4, 583 | 1, 275 | 3, 405 |

Q-GRFT (n=11) and placebo (n=4). Abbreviations: BL, baseline; PA, post application; EP, epithelial; LP, lamina propria. *Statistical difference was seen p=0.02; Mann Whitney test.
